# Evaluation of Vaccination Coverage and Compliance Among Medical Students: A Cross-Sectional Documentary Analysis

**DOI:** 10.7759/cureus.117862

**Published:** 2026-10-13

**Authors:** Beatriz Araújo Gonçalves Coelho, Luiza Letti Ferronatto, Pedro Enzo Camargo Luz, Rodrigo dos Santos Lima

**Affiliations:** 1 Medicine, Centro Universitário de Brasília, Brasília, BRA

**Keywords:** healthcare workers, immunization, medical students, occupational health, vaccination coverage, vaccine-preventable diseases

## Abstract

Introduction: Vaccination is a cornerstone of primary healthcare, particularly critical for medical students and healthcare professionals due to constant occupational exposure to infectious agents. This study aimed to evaluate the vaccination status of medical students at a private higher education institution in the Federal District, Brazil, comparing their self-perceived protection with actual immunization records.

Methods: A cross-sectional, descriptive study was conducted between August 2024 and July 2025, involving 90 medical students from various semesters. Data collection utilized structured online questionnaires and documentary analysis of digital vaccination cards submitted via the "Meu SUS Digital" platform or physical records. Evaluated immunizations included hepatitis A, hepatitis B (HBV), measles, mumps, and rubella (MMR), tetanus/dT, yellow fever, meningococcal C and ACWY, influenza (2024 campaign), and COVID-19. Data were analyzed using absolute and relative frequencies.

Results: Although 75 (83.3%) participants self-reported having an up-to-date vaccination card and 85 (94.4%) felt protected for clinical practice, documentary analysis revealed severe gaps. Only 16 (19.3%) participants possessed a completely up-to-date occupational schedule for all analyzed immunogens. Significant deficits included 56 (67.5%) lacking hepatitis A doses, 77 (92.8%) lacking meningococcal ACWY records, and 22 (26.5%) lacking the 2024 influenza vaccine. The primary reasons cited for missing vaccines were lack of availability at the appropriate age (n = 10, 43.5%) and unknown causes (n = 10, 43.5%). Descriptively, students with parents in the healthcare field and those in advanced internship semesters showed proportionally higher completeness rates, although overall coverage remained insufficient. Furthermore, 57 (63.3%) respondents recognized a lack of information regarding occupational infection risks during their academic training.

Conclusions: A profound discrepancy exists between medical students' perceived immunological protection and their actual immunization status. These findings highlight an urgent need for institutional interventions, curriculum enhancements, and mandatory vaccination verification protocols to safeguard students and patients in clinical learning environments.

## Introduction

Vaccination represents one of the most effective and safe preventive interventions, playing a vital role in preventing the spread of infectious diseases [1]. Healthcare professionals and medical students frequently encounter individuals affected by vaccine-preventable diseases, making proper immunization essential to mitigate occupational risks [2]. Occupational hazards affect millions of workers globally, with infectious diseases representing a major concern among healthcare personnel due to exposure to bloodborne pathogens such as hepatitis B (HBV), hepatitis C (HCV), and human immunodeficiency virus (HIV) [1].

Medical students are particularly vulnerable during their clinical rotations due to early hospital exposure, variable compliance with universal biosafety measures, and learning-associated stress [3]. Vaccination in this context serves a dual purpose: individual protection and the prevention of nosocomial transmission to vulnerable patients, peers, and family members [2]. In Brazil, the Unified Health System (SUS), through the National Immunization Program (PNI), provides free immunizations across all age groups, including specific recommendations for healthcare workers [4].

Despite established guidelines, compliance failures among medical students are frequently documented, resulting in inadequate coverage and heightened occupational exposure [5]. Many students remain unaware of their true vaccination status, compromising safe and ethical clinical practice [6,7]. This study aimed to describe the vaccination status of medical students at a private university, identifying the frequency of unvaccinated individuals according to PNI guidelines and exploring contributing factors to incomplete immunization schedules.

## Materials and methods

Study design and setting

This research was characterized as an applied study with a quantitative approach and a cross-sectional, descriptive design. It combined documentary analysis of vaccination cards with structured questionnaires administered to medical students. The study was conducted at a private higher education institution in the Federal District, Brazil.

The target population comprised medical students regularly enrolled between August 2024 and July 2025. Although the Institutional Review Board approved a maximum sample size of 200 participants, the final convenience sample consisted of 90 students. Inclusion criteria required participants to be 18 years or older, regularly enrolled, and willing to complete the questionnaire and submit vaccination records. Obtaining informed consent was a mandatory prerequisite for enrollment in the study. Seven participants who had lost their physical vaccination documents were retained in the overall sample (N = 90) for the questionnaire analysis but were strictly excluded from the documentary analysis, yielding a precise subsample of 83 valid records for coverage evaluation.

Data collection instruments

Data collection was executed using Google Forms (Google, Inc., Mountain View, CA) (Appendix A). The primary instrument gathered sociodemographic data, vaccination history, and self-perceived immunization status, alongside digital uploads of official vaccination records (from the "Meu SUS Digital" platform or physical cards). A secondary, restricted-access form was used by researchers to tabulate specific doses for each vaccine of interest (hepatitis A, hepatitis B, meningococcal C, meningococcal ACWY, COVID-19, measles, mumps, and rubella (MMR), yellow fever, dT, and influenza 2024), evaluated against National Immunization Program (PNI) 2024 standards (Appendix B).

Given the descriptive nature of the study design, quantitative data were analyzed using Microsoft Excel version 16.0 (Microsoft Corp., Redmond, WA) strictly for descriptive analysis, including absolute and relative frequencies. No inferential statistical tests were applied to determine significance. The study complied with ethical standards established by National Health Council Resolution No. 466/2012 [8] and was approved by the Research Ethics Committee of Centro Universitário de Brasília (CEUB) (CAAE: 82550024.5.0000.0023; approval protocol number: 7.207.863). All participants provided informed consent prior to data collection, confirming their awareness and agreement via an electronic informed consent form (Appendix C).

## Results

Participant profile

Participants were distributed across clinical semesters (36 (40%)), internship phase (35 (38.9%)), and basic science cycles (19 (21.1%)), with ages spanning 18-44 years (mean age: 24.0 ± 4.2 years). Among respondents, 33 (36.7%) reported having at least one parent working in the healthcare sector. Regarding the history of vaccine-preventable diseases, the most prevalent reports were influenza infection in 59 (65.6%), COVID-19 infection post-immunization in 41 (45.6%), and viral gastroenteritis in 41 (45.6%).

Self-perceived immunization status

When asked about their vaccination status relative to the National Immunization Calendar [3], 75 (83.3%) participants believed their schedule was complete, 13 (14.4%) considered it incomplete, and 2 (2.2%) were uncertain. Among the participants who actually reported having missing vaccine doses (n = 23), the main justifications provided were vaccine unavailability for their age group at the time for 10 (43.5%) and a lack of knowledge regarding the reason for 10 (43.5%). Furthermore, 85 (94.4%) participants reported feeling protected when entering hospital environments.

Documentary analysis of vaccination

Card verification of the actual vaccination records submitted by the participants (83 valid cards analyzed, as 7 students reported losing their physical documents) revealed substantial immunization gaps across the evaluated immunogens, as detailed in Table 1.

**Table 1:** Documented vaccination status of medical students across evaluated immunogens (n = 83) The table displays the absolute and relative frequencies of vaccine doses registered on the students' official vaccination cards. Seven participants were excluded from this specific analysis due to documented loss of physical records. Note: The required number of doses for complete immunity varies by vaccine (e.g., hepatitis B requires three doses, whereas yellow fever requires one dose). This table displays the absolute count of documented doses per participant and does not inherently reflect complete immunization for all listed vaccines.

Vaccine type	0 doses	1 dose	2 doses	3+ doses
Hepatitis A	56 (67.5%)	9 (10.8%)	18 (21.7%)	-
Hepatitis B	6 (7.2%)	3 (3.6%)	4 (4.8%)	70 (84.3%)
MMR	7 (8.4%)	2 (2.4%)	74 (89.2%)	-
Influenza (2024 campaign)	22 (26.5%)	61 (73.5%)	-	-
COVID-19	6 (7.2%)	2 (2.4%)	7 (8.4%)	68 (81.9%)
Tetanus (dT/dTpa)	3 (3.6%)	5 (6.0%)	7 (8.4%)	68 (81.9%)
Yellow fever	8 (9.6%)	35 (42.2%)	40 (48.2%)	-
Meningococcal C	48 (57.8%)	35 (42.2%)	-	-
Meningococcal ACWY	77 (92.8%)	6 (7.2%)	-	-

The overall distribution of vaccination records by immunological target is presented in Figure 1. Vaccination compliance was defined strictly according to the universal National Immunization Program (PNI) schedule for healthcare workers. When applying these criteria to the documented records (n = 83), only 16 (19.3%) participants fulfilled the proposed criteria for complete coverage.

**Figure 1:**
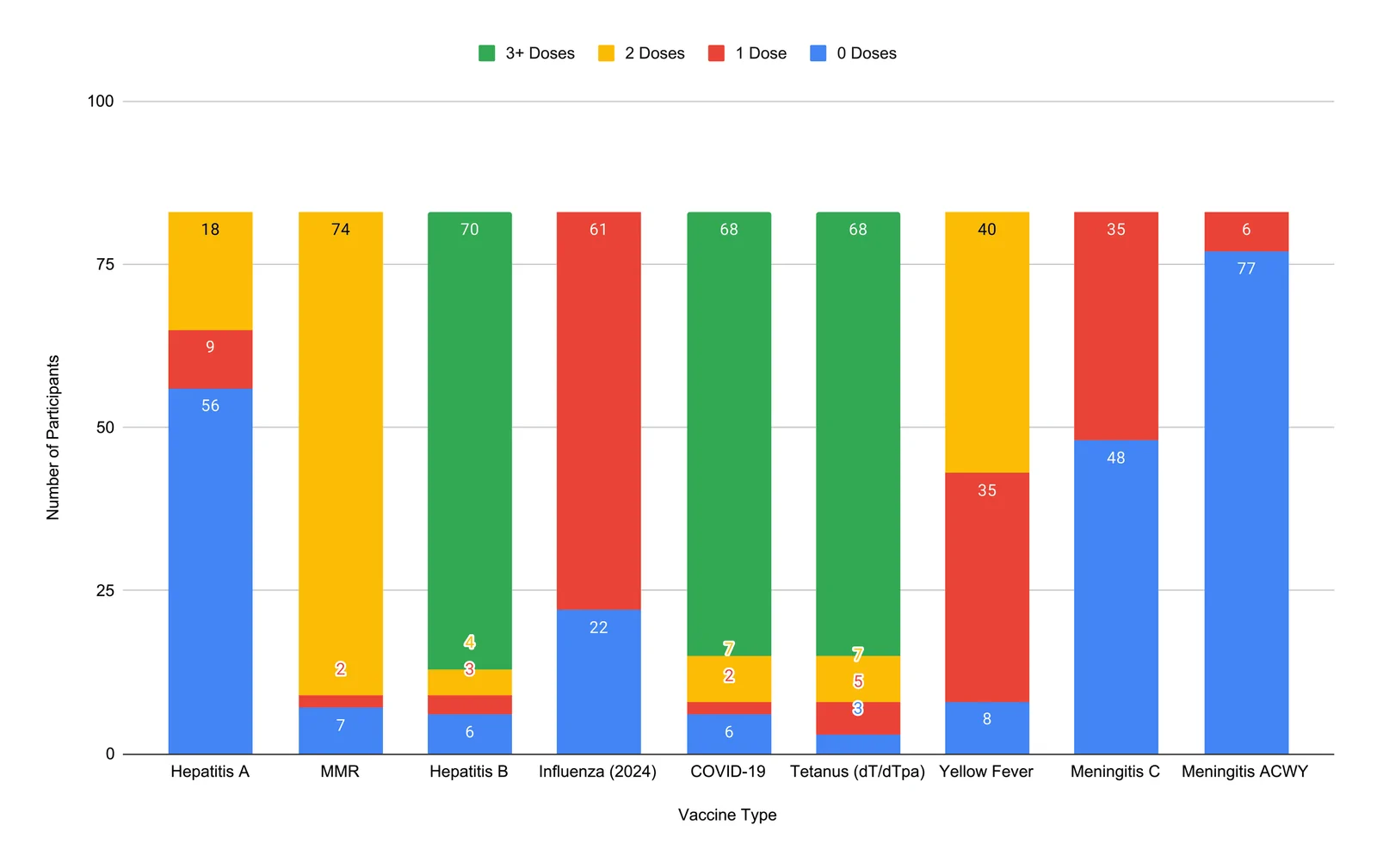
Overall distribution of vaccination records (n = 83) by immunological target The bar chart represents the breakdown of absolute doses administered per vaccine type found during the documentary analysis of the participants' vaccination cards (n = 83). The colors indicate the number of doses received for each specific vaccine.

Discrepancies between perception and reality

Among the participants who self-declared possessing a complete vaccination card, only 15 (20%) actually presented records in full compliance with the recommended schedule for healthcare workers. Conversely, among the 85 students who reported feeling protected to practice in clinical settings, 70 (82.4%) had an incomplete schedule for at least one essential vaccine.

Descriptively, students with at least one parent working in healthcare presented a higher absolute proportion of complete occupational vaccination coverage, being 8 (24.2%) of 33 students, compared to the remaining students, which were 8 (14.0%) of 57 students. When stratified by academic stage, complete occupational vaccination was observed in 7 (20%) internship students, 7 (19.4%) students in the clinical cycle, and 2 (10.5%) students in the basic cycle. As this study relied solely on descriptive frequency analysis, no statistical correlation tests were applied to confirm a significant trend for these differences.

## Discussion

The findings of this study demonstrate a profound discrepancy between medical students' perceived immunological protection and the reality documented in their vaccination cards. This mismatch is alarming, given that clinical trainees face continuous exposure to infectious agents and risk acting as vectors for vulnerable patients. National and international literature consistently highlights suboptimal vaccination coverage among healthcare students [1,4].

In Brazil, the PNI recommends specific occupational schedules for healthcare workers, encompassing hepatitis B, MMR, dTpa, influenza, COVID-19, and yellow fever [9]. While the SBIm also includes vaccines such as meningococcal ACWY for specific risk groups, our universal compliance reference was based strictly on the PNI. Our results highlight severe sub-notification and low coverage, particularly for hepatitis A (56 (67.5%), with zero doses) and meningococcal ACWY (77 (92.8%), with zero doses). The low uptake of the meningococcal ACWY vaccine is largely explained by its recent incorporation into public immunization schedules [10]. However, the finding that 22 (26.5%) students lacked the 2024 annual influenza booster is highly concerning, falling well below the 90% target established by the Ministry of Health [11]. Annual influenza vaccination among healthcare workers is essential, as observational evidence indicates that vaccinated professionals have a 61% lower chance of developing flu-like syndromes compared to unvaccinated peers [12].

Descriptively, students with parents in healthcare exhibited higher complete coverage rates (8 (24.2%) out of 33) compared to others (8 (14%) out of 57), suggesting a potential descriptive trend where familial guidance and health literacy may act as facilitating factors. Similarly, students in advanced semesters (internship phase) demonstrated better compliance (7 (20%) out of 35) than basic science students (2 (10.5%) out of 19), suggesting a descriptive trend where practical clinical exposure might be associated with heightened awareness of occupational hazards. Nevertheless, an overall completion rate of 19.3% (16 out of 83) remains critically insufficient for individuals frequently exposed to immunopreventable pathogens.

Limitations

This study has limitations that should be acknowledged. The sample size was relatively small (N = 90) and restricted to a single private institution, limiting the generalizability of the findings. Reliance on physical cards or the "Meu SUS Digital" platform may not capture vaccines administered in private clinics that were not properly digitized, potentially underestimating actual coverage. Finally, the study design was strictly descriptive; therefore, no inferential statistical analyses were performed to confirm significant associations between sociodemographic variables and vaccination compliance.

These results underscore an institutional responsibility. Medical schools must implement proactive strategies, such as mandatory card verification upon clinical transition, internal vaccination campaigns, and curriculum enrichment addressing occupational biosecurity.

## Conclusions

This study highlights a critical vulnerability in the occupational health and safety of future physicians. Although most medical students exhibited high confidence in their personal immunological protection and believed their vaccination records were complete, documentary analysis revealed pervasive and severe deficits across essential occupational immunizations. The profound mismatch between self-perceived safety and actual documented coverage underscores a systemic failure in health literacy regarding biosecurity during undergraduate medical training. To mitigate the risk of trainees acting as susceptible hosts and potential vectors in clinical environments, medical institutions must transcend passive recommendations. The implementation of proactive institutional policies, such as mandatory vaccination record auditing prior to clinical rotations, targeted educational interventions on occupational hazards, and facilitated access to required immunizations, is imperative to safeguard both the students and the vulnerable patient populations they serve.

## Appendices

Appendix A

Table 2 presents the questionnaire used in the present study.

**Table 2:** Questionnaire for the evaluation of vaccination coverage among medical students at a private institution

Section	Question/item	Response options
Section 1: Identification	Full name	(Free text)
	Date of birth	(Free text)
	Declaration of minimum age of 18 years	( ) Yes ( ) No
	Current semester in medical school	(Free text)
	Are your parents healthcare professionals?	( ) Yes ( ) No
	Maternal education level	( ) Illiterate ( ) Completed elementary school ( ) Completed high school ( ) Completed higher education
	Paternal education level	( ) Illiterate ( ) Completed elementary school ( ) Completed high school ( ) Completed higher education
	Have you ever contracted any vaccine-preventable disease? (Select all that apply.)	( ) Tuberculosis ( ) Hepatitis B ( ) Diphtheria ( ) Tetanus ( ) Pertussis ( ) Poliomyelitis ( ) Pneumonia (pneumococcus) ( ) Pneumonia (other) ( ) Meningitis ( ) Viral gastroenteritis ( ) Mild influenza ( ) Severe influenza ( ) Mild COVID-19 (unvaccinated) ( ) Severe COVID-19 (unvaccinated) ( ) Mild COVID-19 (vaccinated) ( ) Severe COVID-19 (vaccinated) ( ) Yellow fever ( ) Measles ( ) Mumps ( ) Rubella ( ) Varicella ( ) Hepatitis A ( ) HPV ( ) None ( ) Other
Section 2: Vaccination card	Submission	( ) Upload immunization card and "Meu SUS Digital" photo ( ) I lost my vaccination card ( ) I wish to meet researchers in person for analysis
	Healthcare network where immunizations were administered	( ) Private network ( ) Public network ( ) Both
Section 3: Student's perspective	Classification of vaccination card according to National Schedule	( ) Complete ( ) Incomplete ( ) I do not know ( ) Other
	Reason for incomplete vaccination (if applicable)	( ) I did not know ( ) Unavailable for my age ( ) Do not believe in efficacy ( ) No interest ( ) Lack of financial resources ( ) Fear of adverse reactions ( ) Consider disease rare/not severe ( ) Other
	Feel protected attending hospital settings?	( ) Yes ( ) No ( ) Other
	Lack of information on infection risks in undergraduate environment?	( ) Yes ( ) No ( ) Other
	Miss receiving instruction about required immunizations?	( ) Yes ( ) No ( ) Other

Appendix B

The form used to tabulate specific doses for each vaccine of interest is shown in Table 3.

**Table 3:** Data extraction form for vaccination card analysis

Vaccine/immunobiological	Number of doses recorded
Hepatitis A	( ) 0 doses ( ) 1 dose ( ) 2 doses
MMR	( ) 0 doses ( ) 1 dose ( ) 2 doses
Hepatitis B	( ) 0 doses ( ) 1 dose ( ) 2 doses ( ) 3 doses
Influenza 2024	( ) 0 doses ( ) 1 dose
COVID-19	( ) 0 doses ( ) 1 dose ( ) 2 doses ( ) 3 doses ( ) 4 doses ( ) 5 doses ( ) 6 doses
Tetanus (DTP/dT)	( ) 0 doses ( ) 1 dose ( ) 2 doses ( ) 3 doses ( ) 3 doses + booster

Appendix C

Informed Consent Form (ICF)

Below is the ICF containing all the information about the research. Your participation is very important for the development of this study.

You are being invited to participate as a volunteer in the study "Evaluation of Vaccination Coverage Among Medical Students at a Private Institution," developed by researchers from the Centro Universitário de Brasília (CEUB). The name of this document you are reading is the Informed Consent Form (ICF), which aims to ensure your rights as a participant.

Your collaboration in this study will be of great importance to us, but if you decide to withdraw at any time, it will not cause you any harm. Before deciding whether you wish to participate (freely and voluntarily), you must read and understand the entire content.

The research aims to describe the vaccination status of medical students at a private university, seeking to identify the frequency of unvaccinated individuals according to the National Immunization Program (PNI) and the factors that contributed to non-vaccination in these cases, in addition to associating sociodemographic variables with the students' vaccination status.

Your participation consists of filling out the form with your identification data, attaching a photo of your vaccination record, and answering the questionnaire at the end. The identification data to be collected includes full name, date of birth, current semester in the program, history of contracting any vaccine-preventable disease listed in the PNI, and maternal and paternal information regarding education level and whether they are healthcare professionals. Furthermore, photos of the physical vaccination card and/or vaccination record from the "Meu SUS Digital" website will be collected. Finally, students will be asked about the reasons for non-vaccination and their opinions on protection and information regarding vaccine-preventable diseases in practical settings.

There will be no cost for you to participate in this study. There is no possibility of physical harm to the participants. Moral damages could potentially occur due to a breach of confidentiality of the obtained information. The study will follow the resolutions of the National Health Council No. 466 of December 12, 2012; Operational Norm No. 001/2013 of September 30, 2013; and the Operational Manual for Research Ethics Committees.

The results obtained from the research will help determine the frequency of unvaccinated individuals who are at risk of contracting infections for which vaccines are available in Brazil. After the questionnaire, participants will be advised to update their vaccination schedule. Through your participation in this research, your vaccination card will be verified according to the current PNI schedule, and you will receive instructions on which vaccines you should take if your record is incomplete, in addition to specific information about the main diseases and complications that may occur due to the lack of the missing immunizations.

Your participation is voluntary. You will not suffer any penalty if you choose not to participate. You may withdraw from this research at any time by simply contacting one of the responsible researchers. It must also be clarified that the participant has the right not to answer any of the questions.

As established by Brazilian regulations for research involving human subjects, you will not receive any financial compensation for your participation in this study.

Your data will be handled exclusively by the researchers, and access by other individuals will not be permitted. The data, questionnaire, and vaccination card will be kept under the responsibility of the researchers, guaranteeing privacy and confidentiality, and will be archived for a period of three years; after this time, they will be destroyed. The results of this work may be presented at conferences or in scientific journals. However, only aggregate results will be shown, without revealing your name, the institution you belong to, or any information related to your privacy.

If you have any questions regarding the objectives, procedures, and methods used in this research, please contact the responsible researchers (contact information redacted for publication). Also, if you have any considerations or questions regarding the ethical aspects of the research, please contact the Research Ethics Committee of the Centro Universitário de Brasília (CEP-UniCEUB), which approved this research (contact information redacted for publication). The CEP-UniCEUB office hours are Monday to Thursday, from 09:30 AM to 12:30 PM and 02:30 PM to 06:30 PM. You may also contact them to report any irregular or harmful occurrences during your participation in the study.

The CEP is an autonomous group of professionals from various fields of knowledge and the community, of public relevance, whose purpose is to defend the interests of research participants in their integrity and dignity and to contribute to the development of research within ethical standards.

( ) I agree to participate in the study presented herein.
